# Serum antioxidant enzymes in spinal stenosis patients with lumbar disc herniation: correlation with degeneration severity and spinal fusion rate

**DOI:** 10.1186/s12891-023-06907-8

**Published:** 2023-10-03

**Authors:** Youfeng Guo, Yu Zhou, Haihong Zhao, Tao Hu, Desheng Wu

**Affiliations:** 1 Department of spine surgery, Shanghai East Hospital, School of Medicine, Tongji University, Shanghai, 200092 China; 2 Department of medical genetics, School of Medicine, Tongji University, Shanghai, 200092 China

**Keywords:** Intervertebral disc degeneration, Superoxide dismutase, Glutathione reductase, Spinal fusion, Severity, Prognosis

## Abstract

**Objective:**

To determine whether superoxide dismutase (SOD) and glutathione reductase (GR) correlated with the intervertebral disc degeneration (IDD) severity and the postoperative spinal fusion rate in lumbar spinal stenosis patients accompanied with lumbar disc herniation.

**Methods:**

This retrospective study investigated 310 cases of posterior lumbar decompression and fusion. The cumulative grade was calculated by adding the pfirrmann grades of all the lumbar discs. Subjects were grouped based on the median cumulative grade. Logistic regression was used to determine the associations among the demographical, clinical, and laboratory indexes and severe degeneration and fusion. The receiver operating characteristic (ROC) curve was performed to measure model discrimination, and Hosmer-Lemeshow (H-L) test was used to measure calibration.

**Results:**

SOD and GR levels were significantly lower in the severe degeneration group (cumulative grade > 18) than in the mild to moderate degeneration group (cumulative grade ≤ 18). Furthermore, the SOD and GR concentrations of the fusion group were significantly higher than that of the non-fusion group (p < 0.001 and p = 0.006). The multivariate binary logistic models revealed that SOD and GR were independently influencing factors of the severe degeneration (OR: 0.966, 95%CI: 0.950–0.982, and OR: 0.946, 95%CI: 0.915–0.978, respectively) and non-fusion (OR: 0.962; 95% CI: 0.947–0.978; OR: 0.963; 95% CI: 0.933–0.994). The models showed excellent discrimination and calibration. Trend analysis indicated that the levels of SOD and GR tended to decrease with increasing severity (p for trend < 0.001 and 0.003). In addition, it also revealed that SOD provided protection from non-fusion in a concentration-dependent manner (p for trend < 0.001). However, GR concentration-dependent effects were not apparent (p for trend = 0.301).

**Conclusion:**

High serum SOD and GR levels are associated with a better fusion prognosis and a relief in degeneration severity.

## Introduction

Intervertebral disc degeneration (IDD) is a common musculoskeletal disease, which is the pathological and physiological process of natural degeneration, and underlies a variety of clinical spinal diseases such as disc herniation, spinal stenosis, and degenerative spondylolisthesis [1]. The disease is a common cause of back pain in patients and may require surgery if conservative treatment fails to improve [2]. Although disc degeneration is prevalent worldwide and causes a high socioeconomic burden, the exact molecular mechanisms remain unclear. It is reported that the main characteristic of IDD is the nucleus pulposus (NP) reduction in the number of cells and extracellular matrix eventually that alters the structure and properties of the intervertebral disc [3, 4]. Although the cause of disc degeneration is still unknown, inflammation and oxidative stress abnormalities are thought to play an essential role in the disease [5, 6]. It is worth mentioning that many enzymes play a significant role in the process of IDD. On the one hand, these enzymes regulate intervertebral disc degeneration through corresponding signaling pathways. On the other hand, the expression of enzymes is also regulated by various internal and external stimuli, hence mediating degeneration through corresponding signaling pathways. For example, Hua et al. found that miR-127-5p imbalance can promote type II collagen degradation by targeting matrix metalloproteinase-13 in the intervertebral disc, causing disc degeneration [7]. However, after the inactivation of matrix metalloproteinase-8, the expression of inflammatory genes in intervertebral disc tissue increased [8]. For another example, Yao et al. found that Liraglutide can activate PI3K/Akt/mTOR/Caspase-3 and PI3K/Akt/GSK3 β/ The caspase-3 signaling pathway protects the nucleus pulposus from high glucose induced cell apoptosis [9]. In addition, the oxidase and antioxidant enzyme expression imbalance in intervertebral disc degeneration have been reported [10]. Therefore, a comprehensive understanding of oxidase and antioxidant enzymes is essential to maximize the development of new treatments to prevent and delay intervertebral disc degeneration.

Oxidative stress may result from the imbalance between oxidative and antioxidant defense systems. In recent years, many studies have reported that oxidative stress plays a crucial role in the occurrence and development of various diseases [11, 12]. Oxidative stress could result in many neutrophils infiltrating, increasing the production of proteases and reactive oxygen species (ROS). Excessive ROS produced both exogenously and endogenously results in cell structure damage and ultimate cell death [13]. At the same time, oxidative stress also increases with age, resulting from cumulative free-radical lesions over time [14]. It is worthwhile to mention that IDD is a progressive age-related disorder in which oxidative stress plays a key role in nucleus pulposus degeneration [15, 16]. Recent studies have shown that ROS regulation can reduce the apoptosis rate of nucleus pulposus cells and delay chondrocyte degeneration [17–19]. Therefore, preventing the risk of oxidative stress and its associated factors may help alleviate IDD.

Superoxide dismutase (SOD) is an influential group of antioxidants widely distributed in all living systems. SOD is specialized in eliminating superoxide ion radicals from extracellular stimulants and can also act synergistically with Glutathione peroxidase (GPx) or Catalase (CAT) to completely remove harmful substances such as hydrogen peroxide [20, 21]. Therefore, it plays a vital role in life activities and is often used to treat cancer, inflammation, autoimmune diseases, and other diseases [22]. Glutathione reductase (GR) is at the core of one of the most important cellular antioxidant systems. GR can clear ROS when it is in its reduced form, thus contributing to the control of redox homeostasis, which is of great value in protecting the biological activities of sulfhydryl proteins and enzymes and the integrity of cell membranes [23]. At the same time, some studies have suggested that IDD is related to the decrease of the above antioxidant enzymes [24, 25].

Additionally, oxidative stress can aggravate bone loss and inhibit osteogenesis while negatively impacting the immune system and skeletal system [26]. Clinically, spinal fusion has become one of the most commonly used orthopedic procedures for degenerative spinal disorders such as scoliosis, spondylolisthesis, or disc herniation. It has been shown that oxidative stress plays a role in spinal fusion processes, contributing to bone degradation and delayed bone healing [27]. Therefore, studies have shown that inhibition of the oxidative stress response can significantly increase bone mass and contribute to spinal fusion.

Through literature retrieval, we found that most of the existing studies on the relationship between antioxidant enzymes and IDD were focused on detecting the content of the above enzymes in degenerative disc tissues but failed to be combined with clinical practice and spinal fusion prognosis [28, 29]. Therefore, this study was designed to explore the potential correlations between serum antioxidant enzymes and degeneration severity and spinal non-fusion and provide clinical validation for basic research.

## Methods

### Study design

This study was a single-center, cross-sectional analysis. All lumbar spinal stenosis patients accompanied with lumbar disc herniation admitted for lumbar fusion surgery to Shanghai East Hospital between June 2020 and December 2021 were included in the study. The inclusion criteria are: low back pain with or without numbness, pain, fatigue, and other symptoms of lower limbs, as well as a diagnosis of lumbar disc herniation (containing only herniated and bulging discs) or lumbar spinal stenosis, planned lumbar fusion surgery. In addition, patients included in the study were required to undergo antioxidant oxidase testing and report their levels in the inpatient record system. A total of 310 patients were enrolled in this study. The last patients in the study were admitted in March 2021 and completed a 2-year follow-up in March 2023.Patients with a prior history of spinal trauma and tumor, various acute and chronic infectious diseases, and connective tissue diseases were excluded from the study. In addition, patients with underlying diseases, such as severe liver or kidney dysfunction, or heart failure that may affect serum antioxidant enzyme levels, were also excluded. At the same time, patients who were not tested for serum antioxidant enzymes or who had been tested but did not have this reported data in the medical record system were also excluded from the study. The program was approved by the Ethics Committee of Shanghai East Hospital. All study participants gave informed consent.

### Data collection

We retrospectively analyzed the medical records of selected patients in our hospital’s database. Demographic characteristics such as sex, age, BMI, comorbidities such as diabetes, and lifestyle habits such as smoking and excessive alcohol consumption may also be considered. All blood samples were collected within 24 h of admission. An automatic biochemical analyzer determined the levels of SOD and GR. Serum lipids and biochemical analyses (including serum creatinine (Scr), uric acid (UA), lactate dehydrogenase (LDH), fasting blood glucose (FBG), homocysteine (Hcy), and ionized calcium) were recorded.

This study assessed disc degeneration on T2-weighted images based on the Pfirrmann classification [30]. In Grade I, the disc structure is uniformly bright white with a normal intervertebral height. Grade II is characterized by unevenly high signals, a clear boundary between the nucleus pulposus and the annulus fibrosus, and a normal height of vertebral space with or without gray horizontal bands. In grade III, the intervertebral disc structure signals are uneven, medium gray, the nucleus pulposus-annulus fibrosus boundary is unclear, and the vertebral space height is normal or slightly reduced. Grade IV: Uneven structural signals of the intervertebral disc, black-gray low signal changes, loss of the boundary between the nucleus pulposus and the annulus fibrosus, normal or moderately reduced vertebral space height. Grade V is characterized by unbalanced structural signals of the intervertebral disc, low black signals, and a loss of the boundary between the nucleus pulposus and the annulus fibrosus, which causes the intervertebral space to collapse. Decompression and fusion surgery were performed on the patients using a conventional posterior surgical approach. All resected discs were found to be responsible during intraoperative fluoroscopy before removal. Surgeries were performed by an experienced spine surgeon. Follow-up radiography was prescribed for the patients after discharge. The imaging system collected lumbar CT and MRI data from patients two years after spinal fusion surgery to assess spinal fusion. All imaging evaluations were performed blindly by two experienced spine surgeons.

### Statistical analysis

All data were processed by SPSS software 26.0. Quantitative variables were expressed as mean ± standard deviation (mean ± SD) or median [first quartile, third quartile], and categorical variables were shown as composition ratio or rate (%). The Shapiro-Wilk test was used to evaluate the normal distribution of quantitative variables. Statistical comparisons of quantitative data between the two groups were conducted by appropriate T-test or rank-sum test. Categorical variables were compared using the Chi-square test or Fisher precision test. Appropriate statistical tests were used to examine the correlations between variables, including Spearman’s rho test and Kendall’s tau test. Bonferroni correction was used for multiple comparison corrections. The receiver operating characteristic (ROC) curve and the area under ROC (AUC) were performed to examine the predictive ability of the SOD and GR levels for severe degeneration and spinal fusion. Binary logistic regression analysis was used to identify independent risk factors for severe degeneration and non-fusion. The predicting power of variables in the final built model was also checked by the ROC curve. Differences between ROC curve AUC values were assessed using DeLong’s test. Logistic regression models were evaluated for the goodness of fit using the Hosmer-Lemeshow (H-L) test. P < 0.05 was considered statistically significant.

## Results

### Study population

The characteristics of 310 patients included in the analysis are presented in Table 1. The median age was 66.50 years, and 56.8% of the cases were females. The mean BMI of the patients was 24.76 kg/m^2^. Some comorbidities, such as osteoporosis and diabetes, were observed in 112 and 53 patients, respectively. The median TG, TC, LDL, HDL, Scr, UA, FBG, Hcy, LDH, and calcium concentrations were 1.48 mmol/L, 4.45 mmol/L, 2.72 mmol/L, 1.26 mmol/L, 67.00 mmol/L, 320.00 mmol/L, 5.15 mmol/L, 10.60 mmol/L, 174 mmol/L, 2.26 mmol/L, respectively. 207 patients (66.8%) achieved successful spinal fusion during follow-up. Age (*p* < 0.001), osteoporosis (*p* < 0.001), TC (*p* = 0.007), LDL (p = 0.019), lumbar CT value (p < 0.001) and Hcy (p = 0.001), SOD (p < 0.001), GR (p = 0.015), and fusion rate (p = 0.007) were statistically significant between high score group (cumulative grade > 18) and low score group (cumulative grade ≤ 18). A significant difference in gender distribution, lifestyle habits (e.g., smoking and heavy drinking), BMI, the incidence of diabetes, VAS, length of hospital stay, or hematological indicators other than TC, LDL, and Hcy were not found between the two groups.

**Table 1:** Demographic characteristics of patients with disc degeneration disease

	All	Low score group	High score group	*p* value
		(Cumulative grade ≤ 18)	(Cumulative grade > 18)	
Subjects, n (%)	310	160(51.6)	150(48.4)	
Age, years	66.50[57.75-72.00]	62.00[50.00–69.00]	69.00[63.00–75.00]	*< 0.001*
Gender				0.673
Male, n (%)	134(43.2)	71(44.4)	63(42.0)	
Female, n (%)	176(56.8)	89(55.6)	87(58.0)	
BMI, kg/m2	24.76 ± 3.51	25.00 ± 3.61	24.51 ± 3.39	0.218
Smoking	39(12.6)	20(12.5)	19(12.7)	0.965
Alcohol abuse	25(8.1)	11(6.9)	14(9.3)	0.427
DM	53(17.1)	25(15.6)	28(18.7)	0.477
Osteoporosis	112(36.1)	43(26.9)	69(46.0)	< 0.001
TG	1.48[1.01–1.65]	1.47[1.12–1.70]	1.48[0.94–1.61]	0.233
TC	4.45[3.88–4.99]	4.55[4.01–5.04]	4.33[3.74–4.95]	0.007
LDL	2.72[2.24–3.15]	2.79[2.36–3.16]	2.55[2.04–3.15]	0.019
HDL	1.26[1.07–1.43]	1.27[1.04–1.40]	1.26[1.09–1.44]	0.542
Scr	67.00[58.00–79.00]	66.00[57.00-78.75]	68.00[59.75-79.00]	0.224
UA	320.00[265.75–369.50]	319.50[265.25-372.75]	320.60[266.75-367.25]	0.896
FBG	5.15[4.66–5.74]	5.08[4.63–5.71]	5.23[4.70–5.77]	0.401
Hcy	10.60[8.90–12.40]	10.20[8.70-11.59]	11.28[9.20-13.03]	0.001
Calcium	2.26[2.21–2.31]	2.26[2.21–2.31]	2.27[2.20–2.31]	0.918
LDH	174.00[154.00-202.00]	172.00[153.00-201.00]	175.00[154.75–203.00]	0.381
CT value	128.15[96.93-159.08]	136.15[105.55–170.30]	114.80[88.23-147.33]	< 0.001
Hospital stay	12.00[9.00–14.00]	11.00[9.00–14.00]	12.00[9.00–15.00]	0.101
VAS	3.00[2.00–5.00]	3.00[2.00–4.00]	3.00[2.00–5.00]	0.078
SOD	159.00[147.00-172.00]	165.00[154.00-176.75]	152.50[140.75–164.00]	< 0.001
GR	60.00[52.00–67.00]	61.00[53.00-68.75]	56.50[51.00–65.00]	0.015
Fusion	207(66.8)	118(73.8)	89(59.3)	0.007

Values are expressed as n (%), median [first quartile, third quartile], or mean ± SD. BMI, body mass index; TG, Triglyceride; TC, Total Cholesterol; LDL, low-density lipoprotein; HDL, high-density lipoprotein; Scr, serum creatinine; UA, uric acid; FBG, fasting blood glucose; Hcy, homocysteine; LDH, lactate dehydrogenase; SOD, superoxide dismutase; GR, glutathione reductase; DM, diabetes mellitus; VAS, visual analogue scale

### Severity classification and cut-off values of SOD and GR

Table 2 illustrates the distribution of individual disc scores in the total population. Overall, L1/2, L2/3, and L3/4 were rated less than 4. In sharp contrast, most L4/5 and L5/S1 scores (34.5% and 39.0%) were more than or equal to 4. In addition, we noticed a slight difference from the total study populations: a majority of the L4/5 discs in the low group had scores under 4, and a majority of the discs in the high group, except L1/2, had scores exceeding or equal to 4. We defined a mean pfirrmann grade of < 4 as mild to moderate degeneration, and a grade of ≥ 4 as severe degeneration [31]. The median SOD concentration in the severe degeneration group (pfirrmann grade ≥ 4) was significantly lower than that in the mild-moderate degeneration group (pfirrmann grade < 4) in Table 3. The only GR concentrations that differed significantly between groups were those for L2/3 and L3/4. The results of correlation analysis showed that SOD was significantly associated with age (*p* < 0.001), osteoporosis (*p* < 0.001), TC (*p* < 0.001), LDL (*p* < 0.001), HDL (*p* = 0.004), Hcy (*p* < 0.001), serum calcium (*p* < 0.001), and lumbar CT value (*p* < 0.001) across all demographic and clinical parameters in Table 4. Additionally, GR was only correlated with gender (*p* = 0.027), BMI (*p* = 0.008), FBG (*p* = 0.039), serum calcium (*p* = 0.006), and LDH (*p* < 0.001).

**Table 2:** The Pfirrmann grading system for lumbar disc degeneration

	1	2	3	4	5
All (n = 310)					
L1/2	0	140(45.2)	88(28.4)	43(13.9)	39(12.6)
L2/3	0	88(28.4)	96(31.0)	67(21.6)	59(19.0)
L3/4	1(0.3)	59(19.0)	107(34.5)	77(24.8)	66(21.3)
L4/5	0	24(7.7)	86(27.7)	107(34.5)	93(30.0)
L5/S1	0	24(7.7)	62(20.0)	103(33.2)	121(39.0)
Low score group (n = 160)					
L1/2	0	118(73.8)	32(20.0)	10(6.3)	0
L2/3	0	86(53.8)	62(38.8)	11(6.9)	1(0.6)
L3/4	1(0.6)	56(35.0)	84(52.5)	19(11.9)	0
L4/5	0	22(13.8)	66(41.3)	60(37.5)	12(7.5)
L5/S1	0	24(15.0)	44(27.5)	65(40.6)	27(16.9)
High score group (n = 150)					
L1/2	0	22(14.7)	56(37.3)	33(22.0)	39(26.0)
L2/3	0	2(1.3)	34(22.7)	56(37.3)	58(38.7)
L3/4	0	3(2.0)	23(15.3)	58(38.7)	66(44.0)
L4/5	0	2(1.3)	20(13.3)	47(31.3)	81(54.0)
L5/S1	0	0	18(12.0)	38(25.3)	94(62.7)

Values are expressed as n (%)

**Table 3:** The relationship between the severity of individual disc degeneration and serum SOD and GR levels

		SOD	GR
L1/2	pfirrmann grade < 4	161.50[150.25–175.00]	60.00[52.00–67.00]
	pfirrmann grade ≥ 4	153.00[137.75-162.25] ^*^	57.00[50.75–65.50]
L2/3	pfirrmann grade < 4	164.50[152.00-176.00]	61.00[53.00–68.00]
	pfirrmann grade ≥ 4	154.00[140.75–162.00] ^*^	55.50[51.00-65.25] ^*^
L3/4	pfirrmann grade < 4	165.00[154.00-176.00]	61.00[[53.00–69.00]
	pfirrmann grade ≥ 4	152.00[141.00-164.00] ^*^	57.00[51.00–65.00] ^*^
L4/5	pfirrmann grade < 4	166.00[152.75–178.00]	61.00[52.00-69.50]
	pfirrmann grade ≥ 4	155.00[144.00-167.00] ^*^	59.00[52.00-66.75]
L5/S1	pfirrmann grade < 4	167.00[157.00-178.00]	61.00[51.00–69.00]
	pfirrmann grade ≥ 4	155.00[143.25-168.75] ^*^	59.00[52.00–66.00]

Data are shown as median [first quartile, third quartile]. SOD, superoxide dismutase; GR, glutathione reductase; * represents statistical significance (p < 0.05) between groups using a Mann-Whitney non-parametric test

### Risk factors analysis models for severe disc degeneration

**Fig. 1:**
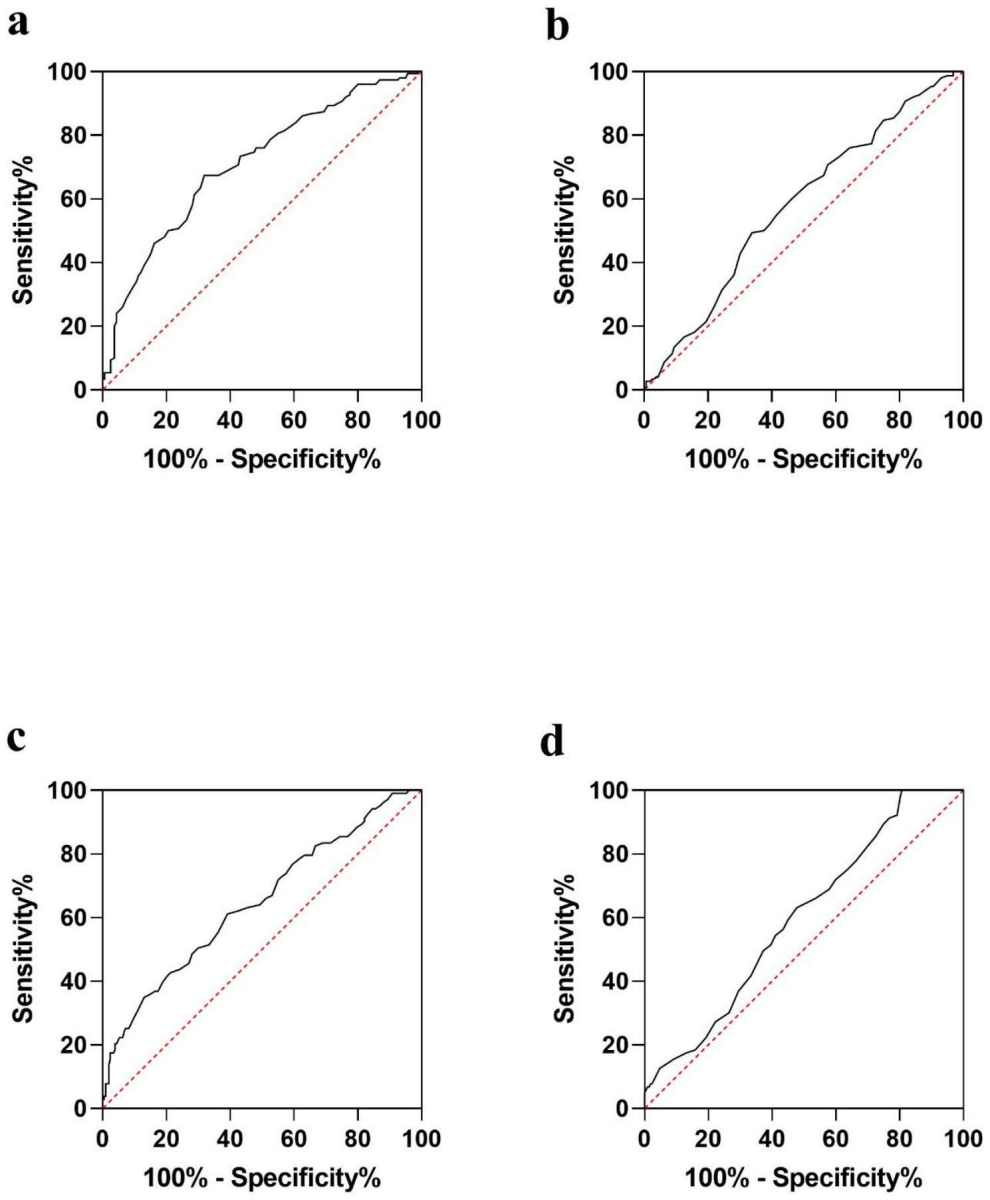
Receiver operating characteristic (ROC) curve to determine the predictive performance of SOD for severe degeneration (**a**) and non-fusion (**c**). Receiver operating characteristic (ROC) curve to determine the predictive performance of GR for severe degeneration (**b**) and non-fusion (**d**)

**Fig. 2:**
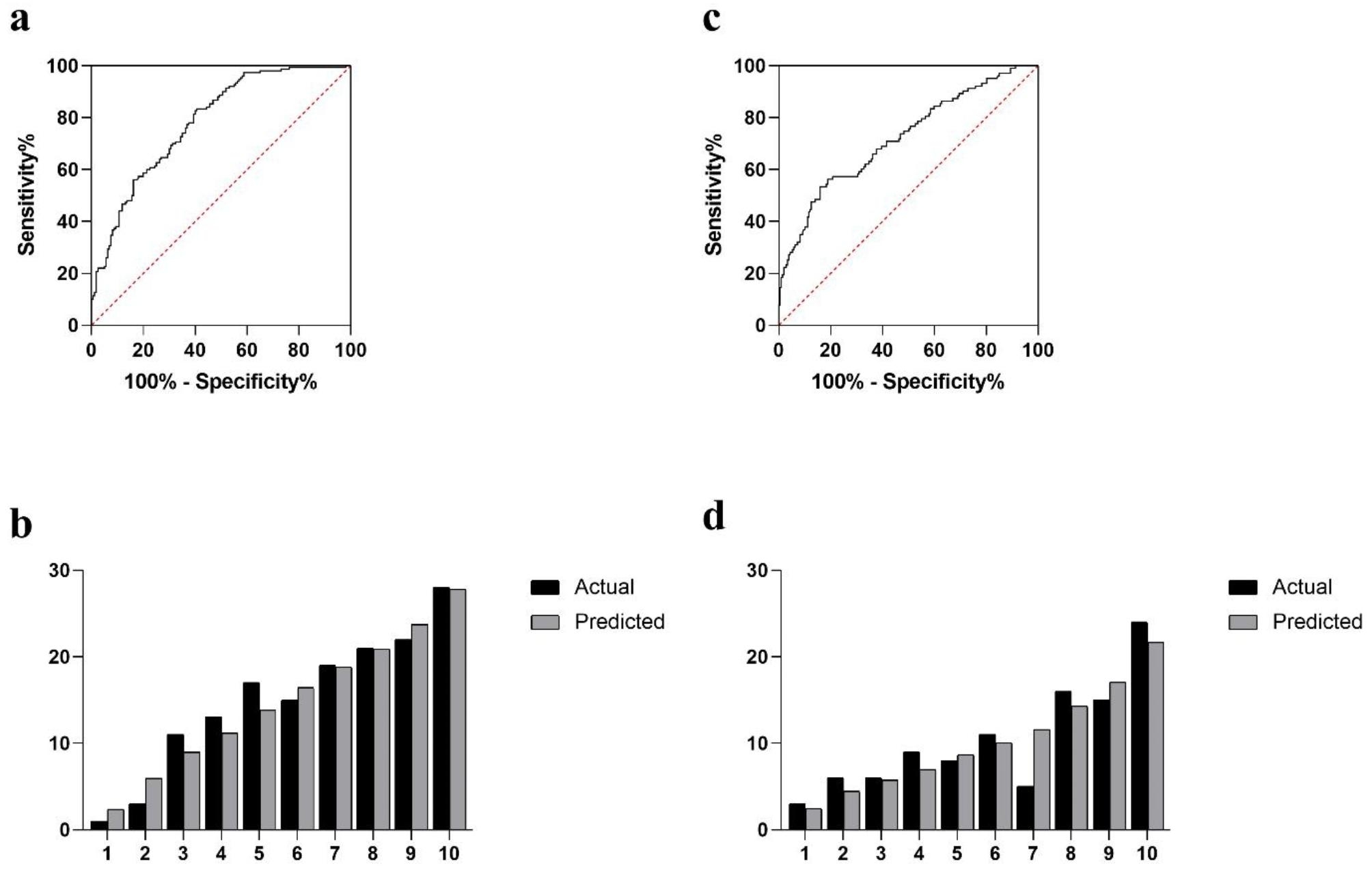
ROC curve analysis of severe degeneration model 3 (**a**) and non-fusion model 6 (**c**) (see Tables 5 and 7 for included variables). Actual versus predicted severe degeneration (**b**) and spinal non-fusion (**d**) by risk deciles for models 3 and 6

**Fig. 3:**
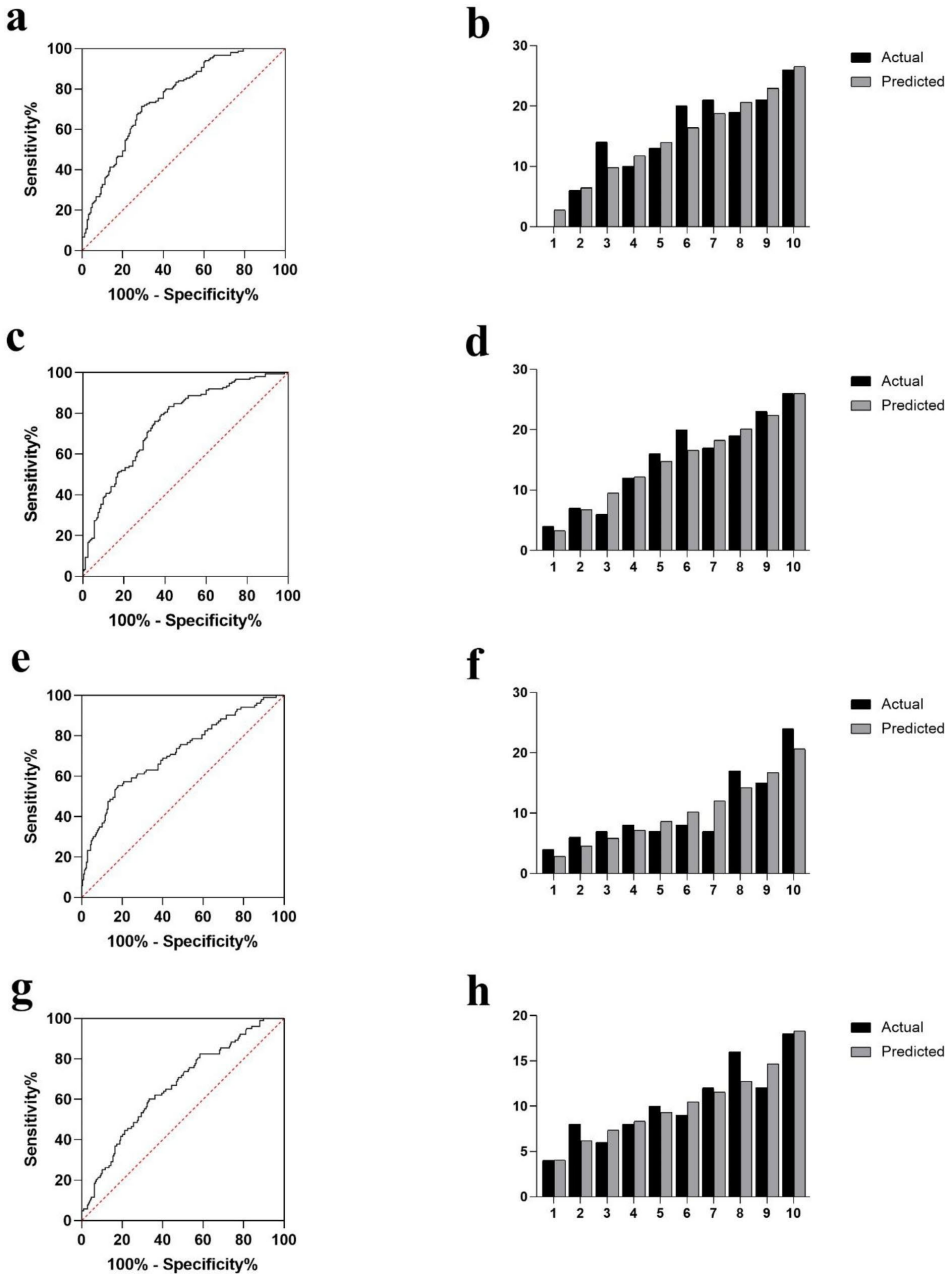
ROC curve analysis of prognostic models 1 and 2 of severe degeneration (**a** and **c**). ROC curve analysis of prognostic models 4 and 5 of spinal non-fusion (**e** and **g**). Actual versus predicted severe degeneration models 1 and 2 (**b** and **d**) and spinal non-fusion models 4 and 5 (**f** and **h**) by risk deciles

GR and SOD were correlated with cumulative grades according to the correlation analysis (p < 0.001 and p = 0.033, respectively). ROC curves were generated to determine whether SOD and GR can predict the severity of disc degeneration. SOD was found to have an area under the curve (AUC) of 0.709 for predicting severe degeneration in Fig. 1a. The larger area under the ROC curve of SOD than that of GR (AUC = 0.580) further suggests the superior capability of SOD to detect severe disc degeneration (Fig. 1a-b). With the SOD and GR cut-off values set at 158.5 and 55.5, the Youden index reaches its maximum value. It was found that each additional unit of age (OR: 1.071, 95%CI: 1.047–1.096), osteoporosis (OR: 2.318, 95%CI: 1.442–3.726), SOD (OR: 0.959, 95%CI: 0.945–0.973), and GR (OR: 0.972, 95%CI: 0.953–0.992) significantly contributed to severe degeneration (Table 5). An analysis of multivariable binary logistic regression based on clinical parameters revealed that every one unit increase in age (OR: 1.059; 95% CI: 1.031–1.088), SOD (OR: 0.966; 95% CI: 0.950–0.982), and GR (OR: 0.946; 95% CI: 0.915–0.978) significantly predicted severe degeneration in the model 3. Trend analysis revealed that SOD and GR provided protection from severe degeneration in a concentration-dependent manner (*p* < 0.001 and *p* = 0.003; Table 6). At the same time, a two-way ANOVA indicated no significant interaction among SOD, GR, and age on severe degeneration. In addition, SOD and GR were equally predictive of severe degeneration in models 1 and 2 (Table 5). The model 3 was significant, with *p* = 0.657 for the Hosmer and Lemeshow goodness of fit test. Meanwhile, in model 3, the observed vs. predicted risk of severe disc degeneration within risk deciles was well matched in Fig. 2b. The area under the ROC curve of model 3 is 0.784 (Fig. 2a). Thus, model 3 performs well in terms of calibration and discrimination (*p* > 0.05 and *p* < 0.05). According to the Delong test of the area under the curve, the model 3 is not significantly different from models 1 and 2 (AUC = 0.756 and 0.758, respectively). The discrimination and calibration of models 1 and 2 are shown in Fig. 3a and d.

**Table 4:** Correlation analysis of SOD, GR, and demographic and clinical parameters

Measures	SOD		GR	
	Correlation (B)	P Value	Correlation (B)	P Value
Age	-0.369	< 0.001	0.063	0.265
Sex (female^*^)	0.026	0.643	-0.126	0.027
BMI	-0.042	0.465	0.151	0.008
DM	-0.006	0.912	0.010	0.857
Osteoporosis	-0.202	< 0.001	0.021	0.713
Smoking	0.077	0.175	-0.090	0.115
Alcohol abuse	0.027	0.639	-0.109	0.055
TG	-0.017	0.760	0.111	0.051
TC	0.203	< 0.001	0.105	0.065
LDL	0.223	< 0.001	0.052	0.360
HDL	0.165	0.004	0.093	0.103
Scr	-0.037	0.513	-0.075	0.191
UA	0.037	0.520	0.063	0.272
FBG	0.070	0.220	0.117	0.039
Hcy	-0.252	< 0.001	0.011	0.852
Calcium	0.294	< 0.001	0.156	0.006
LDH	-0.007	0.897	0.627	< 0.001
CT value	0.207	< 0.001	-0.093	0.103
Hospital stay	-0.054	0.343	0.079	0.164
VAS	-0.041	0.477	0.013	0.825

BMI, body mass index; TG, Triglyceride; TC, Total Cholesterol; LDL, low-density lipoprotein; HDL, high-density lipoprotein; Scr, serum creatinine; UA, uric acid; FBG, fasting blood glucose; Hcy, homocysteine; LDH, lactate dehydrogenase; SOD, superoxide dismutase; GR, glutathione reductase; DM, diabetes mellitus; VAS, visual analogue scale

**Table 5:** Univariate and multivariate analysis models of risk factors for severe degeneration

Variable	Univariate	Multivariate		
		Model 1	Model 2	Model 3
SOD	0.959(0.945–0.973)		0.965(0.950–0.980)	0.966(0.950–0.982)
GR	0.972(0.953–0.992)	0.942(0.913–0.973)		0.946(0.915–0.978)
Age	1.071(1.047–1.096)	1.071(1.044–1.099)	1.054(1.026–1.082)	1.059(1.031–1.088)
Sex (female^*^)	0.908(0.579–1.423)	1.021(0.517–2.015)	1.145(0.576–2.277)	1.118(0.555–2.249)
BMI	0.961(0.901–1.024)	0.999(0.924–1.080)	0.975(0.902–1.054)	0.990(0.913–1.073)
DM	1.239(0.685–2.241)	0.963(0.502–1.845)	1.111(0.575–2.147)	1.085(0.556–2.116)
Osteoporosis	2.318(1.442–3.726)	1.105(0.614–1.988)	1.116(0.615–2.026)	1.042(0.565–1.919)
Smoking	1.015(0.519–1.987)	1.199(0.504–2.853)	1.202(0.496–2.912)	1.217(0.491–3.014)
Alcohol abuse	1.394(0.612–3.176)	0.746(0.280–1.987)	1.131(0.425–3.014)	0.854(0.306–2.387)
LDL	0.770(0.577–1.028)	0.911(0.652–1.273)	1.011(0.718–1.425)	1.028(0.724–1.461)
HDL	1.348(0.677–2.684)	1.250(0.542–2.884)	1.943(0.810–4.663)	1.890(0.769–4.646)
UA	0.999(0.997–1.003)	1.002(0.998–1.005)	1.001(0.998–1.005)	1.002(0.998–1.006)
Hcy	1.014(0.981–1.048)	0.991(0.962–1.021)	0.990(0.961–1.021)	0.985(0.956–1.015)
LDH	1.002(0.996–1.008)	1.009(0.999–1.018)	0.998(0.990–1.005)	1.008(0.998–1.018)

BMI, body mass index; LDL, low-density lipoprotein; HDL, high-density lipoprotein; UA, uric acid; Hcy, homocysteine; LDH, lactate dehydrogenase; SOD, superoxide dismutase; GR, glutathione reductase; DM, diabetes mellitus

**Table 6:** Association of severe degeneration and spinal fusion failure with SOD and GR

Variable	Cases	Model 3		Model 6	
		OR [95%CI]	*p* for trend	OR [95%CI]	*p* for trend
SOD (median[range])					
Q1 (137[≤ 147])	80	Reference		Reference	
Q2 (154[147–159])	79	0.390[0.183–0.830]		0.332[0.161–0.685]	
Q3 (166[159–172])	75	0.288[0.135–0.615]		0.310[0.147–0.656]	
Q4 (180.5[> 172])	76	0.173[0.076–0.393]	< 0.001	0.204[0.090–0.465]	< 0.001
GR (median[range])					
Q1 (48[≤ 52])	86	Reference		Reference	
Q2 (55[52–60])	78	0.849[0.403–1.790]		1.691[0.844–3.391]	
Q3 (64[60–67])	74	0.464[0.209–1.029]		1.271[0.585–2.761]	
Q4 (74[> 67])	72	0.289[0.116–0.722]	0.003	0.668[0.267–1.676]	0.301

SOD, superoxide dismutase; GR, glutathione reductase

**Table 7:** Univariate and multivariate analysis models of risk factors for spinal fusion failure

Variable	Univariate	Multivariate		
		Model 4	Model 5	Model 6
SOD	0.969(0.956–0.983)	0.961(0.946–0.976)		0.962(0.947–0.978)
GR	0.959(0.938–0.982)		0.956(0.928–0.986)	0.963(0.933–0.994)
Age	1.006(0.988–1.024)	0.981(0.959–1.004)	1.001(0.980–1.022)	0.983(0.960–1.006)
Sex (female^*^)	0.914(0.566–1.474)	0.882(0.442–1.759)	0.798(0.409–1.558)	0.862(0.430–1.729)
BMI	0.976(0.912–1.044)	0.967(0.895–1.045)	0.997(0.925–1.074)	0.977(0.903–1.056)
DM	0.758(0.395–1.453)	0.850(0.414–1.743)	0.741(0.372–1.478)	0.846(0.411–1.745)
Osteoporosis	1.621(0.997–2.636)	1.581(0.852–2.935)	1.595(0.875–2.908)	1.488(0.795–2.786)
Smoking	1.006(0.493–2.050)	1.124(0.454–2.781)	1.141(0.484–2.691)	1.145(0.459–2.854)
Alcohol abuse	1.143(0.487–2.683)	1.092(0.405–2.946)	0.745(0.278–2.002)	0.859(0.302–2.449)
LDL	1.038(0.769–1.402)	1.197(0.840–1.707)	1.037(0.742–1.450)	1.213(0.848–1.737)
HDL	1.408(0.684–2.898)	2.905(1.206–6.995)	1.691(0.737–3.880)	2.734(1.130–6.613)
UA	1.001(0.999–1.004)	1.003(0.999–1.007)	1.003(0.999–1.006)	1.004(0.999–1.007)
Hcy	1.029(0.987–1.073)	1.024(0.975–1.076)	1.039(0.979–1.102)	1.021(0.972–1.074)
LDH	0.993(0.986-1.000)	0.990(0.982–0.998)	0.998(0.989–1.008)	0.996(0.987–1.006)

BMI, body mass index; LDL, low-density lipoprotein; HDL, high-density lipoprotein; UA, uric acid; Hcy, homocysteine; LDH, lactate dehydrogenase; SOD, superoxide dismutase; GR, glutathione reductase; DM, diabetes mellitus

### Influencing factors analysis models for spinal fusion

In the correlation analysis, it was found that non-fusion was correlated with both SOD and GR (*p* < 0.001 and *p* = 0.006, respectively). At the same time, the SOD and GR concentrations of the fusion group were significantly higher than that of the non-fusion group (*p* < 0.001 and 0.006). We then calculated the ROC curve to determine if SOD and GR can predict non-fusion. In this study, the area under the curve (AUC) for SOD was found to be 0.647 in Fig. 1c. According to Fig. 1c-d, SOD provides a superior performance in detecting non-fusion than GR, based on its larger area under the ROC curve (AUC = 0.597). The maximum value of the Youden index is reached when SOD and GR cut-off values are 157.5 and 72.5, respectively. A univariate binary logistic regression analysis revealed that GR (OR: 0.959, 95%CI: 0.938–0.982) and SOD (OR: 0.969, 95%CI: 0.956–0.983) are significantly associated with severe degeneration in Table 7. The multivariable binary logistic regression in model 6 based on the clinical parameters also demonstrated that an increase in both SOD (OR: 0.962; 95% CI: 0.947–0.978) and GR (OR: 0.963; 95% CI: 0.933–0.994) was an independent predictor of non-fusion in the model 6. Moreover, trend analysis revealed that SOD provided protection from spinal fusion in a concentration-dependent manner (*p* < 0.001; Table 6). In contrast, no GR concentration-dependent effects were apparent (*p* = 0.301). At the same time, a two-way ANOVA indicated no significant interaction among SOD, GR, and age on non-fusion. Similarly, models 4 and 5 have the same trend as model 6. The model 6 was significant, with *p* = 0.306 for the Hosmer-Lemeshow goodness of fit test. Additionally, within each risk decile, a good fit was observed between the observed and predicted risk of severe disc degeneration in Fig. 2d. The area under the ROC curve of model 6 is 0.721 (Fig. 2c). In this sense, model 6 has effective calibration and discrimination (*p* > 0.05 and *p* < 0.05). The Delong test of the area under the curve shows that the test efficiency of model 6 is significantly different from model 4 (AUC = 0.663) but not from that of model 5 (AUC = 0.715). The discrimination and calibration of models 4 and 5 are shown in Fig. 3e h.

## Discussion

Currently, studies have shown local oxidation to be crucial in the context of degenerative intervertebral discs and osteogenic properties. Song et al. found that the content of antioxidant enzymes such as SOD and GR decreased in the degenerative disc tissue, suggesting that a local imbalance of antioxidant enzymes and oxidase may be related to IDD [28]. At the same time, oxidative stress inhibits osteoblast proliferation and differentiation while reducing oxidative intermediate production stimulates osteogenesis [32]. However, much less attention has been given to the relationship between antioxidant enzymes in circulation and IDD and postoperative spinal fusion rate. As part of our research program, we investigated the correlation among them and whether they have predictive value for the above events. In this study, we found that the levels of SOD and GR in the circulation were positively correlated with spinal fusion; a high concentration of antioxidative enzymes was a contributor to spinal fusion. Further, we also observed that higher SOD and GR were associated with a lighter degeneration.

Because of the dysfunction of the pro-oxidation-antioxidant system, ROS continues to be excessively released, leading to an excessive inflammatory response [33]. Our previous study showed that cytokines such as serum IL-6 and TNF-α concentrations were positively correlated with the severity of IDD [34]. Similarly, the present results reveal that IDD tended to be more severe with a gradual decrease in SOD concentrations. In this study, we found that a low level of SOD was an independent risk factor for severe disc degeneration. In addition, SOD levels were significantly lower in the severe degeneration group than in the mild-to-moderate degeneration group for an individual disc. Although the changes in SOD levels are not surprising, this is the first time to report a positive correlation between low SOD levels in circulation and the severity of IDD in the human body. The same applies to the effect of GR on disc degeneration. Not least, the influence of SOD and GR on severe disc degeneration presented a concentration-dependent pattern. At the same time, high SOD and GR levels are protective factors for postoperative non-fusion. However, SOD showed a concentration-dependent protective effect against non-fusion, while GR did not. Additionally, while SOD had a higher predictive power for non-fusion than GR, this was reversed in the prediction model. This may be related to the clinical and demographic parameters included. The inclusion of SOD did not improve the predictive power of the GR forecast model. Additionally, two markers considered together to assess severe disc degeneration did not add substantial prognostic power to respectively existing risk prediction models. Overall, SOD and GR have some predictive power for spinal fusion and degeneration severity, according to this study. Increasing levels of SOD or GR can alleviate oxidative stress of intervertebral disc tissue and help treat IDD at this point. Xiao et al. found that adipose-derived mesenchymal stem cells modified by the antioxidant SOD2 can improve the histopathologic status of the intervertebral disc, reduce inflammation, and have a therapeutic effect on IVD [35]. In addition, it is likely that drugs or food may be able to alleviate disc degeneration, such as by increasing antioxidant enzyme levels or inhibiting oxidative stress levels [36, 37].

Moreover, there are a few other factors that might have an impact on severe degeneration and non-fusion. For example, some studies have indicated that uric acid is an antioxidant that reduces the production of oxidation products. Under certain circumstances, uric acid leaves the body with oxidative stress due to its limited ability to scavenge free radicals and can destroy the redox balance system in various ways [38]. Moreover, HDL has been shown to have strong antioxidant and anti-inflammatory properties, which may be able to regulate the oxidation state of systemic circulation and blood vessel walls and protect organ function in vivo [39]. However, our failure to find significant effects of them on severe disc degeneration and non-fusion may relate to the small sample size. Additionally, age presents one of the most significant factors that affect the disc degeneration. Previous studies have reported that the degree of IDD and lumbar facet joints degeneration are positively correlated with age [40]. The results of the logistic analysis suggested that age was a risk factor for severe disc degeneration. At the same time, it has been reported that the risk of refusion surgery due to non-fusion increases with age [41]. The reason for this may be due to age-related bone loss caused by decreased osteogenesis with age [42]. We did not observe a significant effect of age on spinal fusion rate in this study, which may be due to the failure to reflect differences in osteogenic ability between different ages in the included population. In addition, demographic parameters such as age, sex and osteoporosis can also affect the level of SOD and GR. For example, the levels of these two enzymes gradually decline with age and are higher in women than in men [43, 44]. In addition, in the oxidative stress environment of osteoporosis, the antioxidant system and oxidation system are dysfunctional, and the level of antioxidant enzymes such as SOD is decreased [45]. Again, the same correlation was found in this experiment. Although there may be inconsistency between the two enzymes and the above parameters, it is not contrary to previous research reports. In terms of blood lipids, we found that SOD level was significantly positively correlated with blood lipids, which was inconsistent with previous studies [46, 47]. A similar situation exists in BMI as well. This may be related to the sample population of this study and may be a compensatory mechanism. In addition, it is worth mentioning that although the diabetic body is full of oxidative stress, there are studies reporting that the level of SOD in diabetic patients is significantly higher than that of normal people [48]. At the same time, the results of this study also suggest that there is a positive correlation between blood glucose and GR. In addition, studies have reported that other components in the blood such as calcium ions can promote the increase and accumulation of SOD content [49, 50], while Hcy has the opposite effect [51].

The current study has several limitations. First of all, it is a retrospective study, with its inherent shortcomings. Second, there may be other factors affecting serum antioxidant enzyme activity, such as diet [52, 53]. Therefore, serum levels of SOD and GR should be dynamically measured. In addition, the diagnosis of comorbidities differs from the judgments of disease severity in the case of the elderly. This calls for a more detailed inquiry about the history of the disease. Moreover, given that the area under the ROC curve in this study was not particularly ideal, we still need to further expand the sample size for long-term follow-up. The practicability of the above cut-off values in other conditions remains further validated. Considering that serum antioxidant enzyme levels may be related to other diseases, such as liver diseases [54], it also provides the direction for our follow-up research. Apart from that, since the relationship between serum antioxidants and the severity of IDD and spinal fusion prognosis has not been reported before, we are unable to calculate the sample size. More samples are needed to validate our findings, particularly to determine whether our findings’ statistical significance is clinically significant. Not only that, because we found in our review of the medical record system that there were cases in which the levels of antioxidant enzymes were not reported in the examination results of some patients, we also plan to cooperate with the clinical laboratory to carry out the corresponding prospective study to ensure that such events can be avoided or reduced.

## Conclusion

In conclusion, we performed a retrospective analysis and found that high SOD and GR levels were associated not only with severe disc degeneration but also with non-fusion. At the same time, the two markers also have somewhat predictive power for the above events.

## Data Availability

The datasets generated and/or analyzed during the present study are available from the corresponding author upon reasonable request.
